# Antimicrobial Property of Extracts of Indian Lichen against Human Pathogenic Bacteria

**DOI:** 10.1155/2013/709348

**Published:** 2013-08-25

**Authors:** Priya Srivastava, D. K. Upreti, T. N. Dhole, Apurva K. Srivastava, Meghanand T. Nayak

**Affiliations:** ^1^Lichenology Laboratory, National Botanical Research Institute (CSIR), Rana Pratap Marg, Lucknow 226001, India; ^2^Department of Microbiology, Sanjay Gandhi Post Graduate Institute of Medical Sciences, Lucknow 226014, India; ^3^Department of Pathology & Microbiology, Saraswati Dental & Medical College, Lucknow 227105, India; ^4^Department of Oral and Maxillofacial Pathology, Vyas Dental College and Hospital, Pali Road, Jodhpur, Rajasthan 342005, India

## Abstract

*Context*. *Usnea ghattensis* G. Awasthi (Usneaceae) endemic fruticose lichen found growing luxuriantly in Northern Western Ghats of India, it also contains Usnic acid as a major chemical and tested against some human pathogenic bacteria. *Objective*. To explore antimicrobial properties of *Usnea ghattensis* against some human pathogenic bacteria. *Materials and Methods*. The lichen was extracted in acetone, methanol, and ethanol. *In vitro* antimicrobial activity was tested initially by *Kirby-Bauer* technique of disc diffusion method and was confirmed by minimum inhibitory concentration using Broth microdilution method according to the NCCLS guidelines. *Results*. Ethanol extract was most effective against *Bacillus cereus* and *Pseudomonas aeruginosa* with a zone of inhibition 29.8 ± 0.6 mm and 12.3 ± 0.5 mm diameters at a concentration of 0.2 mg/mL. Acetone and methanol extract demonstrated almost similar activity against *Staphylococcus aureus* and the zone of inhibition was 24.6 ± 0.5 and 24.7 ± 0.4 mm. Only methanol extract was showing activity against *Streptococcus faecalis* with a 13.5 ± 0.8 mm zone. MIC value noted against *Staphylococcus aureus* and *Streptococcus faecalis* was 6.25 **μ**g/mL and 25 **μ**g/mL, whereas against *Bacillus cereus* and *Pseudomonas aeruginosa*, MIC calculated was 3.125 **μ**g/mL and 200 **μ**g/mL, respectively. *Conclusion*. The present study demonstrates the relatively higher activity of this lichen against not only gram (+) but significantly also against gram (−) bacteria. This indicates that this lichen might be a rich source of effective antimicrobial agents.

## 1. Introduction

Medicinal plants are well-known natural sources for the treatment of various diseases since ancient times. Lichens are among the most fascinating organisms on this planet. Lichen is not a single organism the way most other living things are, but rather it is a combination of two organisms which live together intimately. The fungus forms a thallus or lichenized stroma that may contain characteristic secondary metabolites in all lichens [1]. Lichens are valuable plant resources and are used as medicines, food, fodder, dyes perfume, spice, and for miscellaneous purposes. The lichen flora is rather poor in the vicinity of industrial areas and big cities [2], as lichens are very sensitive to various air pollutions. Thus, these organisms are used as air pollution monitors [3]. The specific, even extreme, conditions of their existence, slow growth, and long duration (maximum lifetime spans to several thousand years) are consistent with their abundance in protective metabolites against different physical and biological influences [4]. Lichens have been used for medicinal purposes throughout the ages, such as *Cetraria islandica* (L.) Ach. (Parmeliaceae), *Lobaria pulmonaria* (Schreb.) Hoffm. (Lobariaceae) were reported to be effective in the treatment of pulmonary tuberculosis [5].

The use of lichens in medicine is based on the fact that they contain unique and varied biologically active substances, mainly with antimicrobial actions. Because of marked antimicrobial activity of secondary metabolites, lichens, macrofungi, and vascular plants attract great attention of investigators as new significant sources of bioactive substances [6–9]. The intensive use of antibiotics has selected for antibiotic resistance factors and facilitated the spread of multiply resistant microorganisms. Lichen metabolites exert a wide variety of biological actions including antibiotic, antimycotic, antiviral, anti-inflammatory, analgesic, antipyretic, antiproliferative, and cytotoxic effects [10–15]. Although about 8% of the terrestrial ecosystem consists of lichens and more than 20,000 lichen species are distributed throughout the world, their biological activities and biologically active compounds remain unexplored to a great extent [16].

*Usnea ghattensis* is an endemic fruticose lichen that grows on different trees and shrubs in Northern Western Ghats of India. Most of the lichen species of the genus *Usnea* containing Usnic acid as the major chemical constituent are used traditionally in upper respiratory infections, and applied on the skin to treat surface infections or external ulcers. Usnic acid has been used as a human papillomavirus (HPV) treatment and as an oral hygiene agent, with limited effectiveness. In accordance with these facts, in this study, the antimicrobial activity of acetone, methanol, and ethanol extracts of *Usnea ghattensis* was investigated *in vitro* in relation to test microorganisms, where some of them promote diseases in humans, animals, and plants and even produce toxins and provoke food deterioration.

## 2. Material and Methods

### 2.1. Microorganisms

Total six bacteria, three gram positive (*Staphylococcus aureus* (ATCC 25923), *Streptococcus faecalis* (ATCC 33186), and *Bacillus cereus* (ATCC 14579)) and three gram negative (*Escherichia coli* (ATCC 25922), *Pseudomonas aeruginosa* (ATCC 29853), and *Salmonella typhimurium* (ATCC 13311)), were used to assess the antimicrobial properties of the test samples. These Bacteria were kept on nutrient agar plates at 4°C, respectively. For use in experiments, the organisms were subcultured in blood agar culture medium and MacConkey's medium.

### 2.2. Lichen Material

The plant material of *Usnea ghattensis* was collected during Dec. 2009 from Lingmala Forest area, Mahabaleshwar, Satara district, Maharashtra, the northern western Ghat area of India between altitudes of 1200 and 1340 m. One voucher specimen was preserved in the herbarium of National Botanical Research Institute, Lucknow (LWG).

#### 2.2.1. Extraction of Lichen Material

The lichen samples were washed to remove debris; the air was dried, pulverized to powder, and stored in a sterile glass bottle in the refrigerator. 10 g portions of sieved powder was added to 100 mL of solvents (acetone, ethanol, and methanol) and left for three days at room temperature. The crude extract was prepared by decanting, followed by filtration through muslin cloth, and further filtered with Whatman No. 1 filter paper to obtain a clear filtrate. The filtrates were further purified by membrane filter using 0.45 *μ*m pore size filters. The extracts were then evaporated to dryness under reduced pressure and redissolved in respective solvents to attain the required concentrations of 0.1 mg/mL and 0.2 mg/mL for antibacterial screening. These extracts were kept at 4°C till used.

### 2.3. Preparation of Antibiotic Disc

Individual crude extracts were dissolved in respective solvents. Two different concentrations of extracts, that is, 0.1 mg/mL and 0.2 mg/mL, were used for preparing disc. Whatman filter paper disc with diameter of 6 mm was used for preparing discs. Each disc was impregnated with 10 *μ*L of lichen's crude extract, allowing the solvent to evaporate between the applications and leaving the lichen extract on discs without the solvent. These freshly prepared discs were used for the determination of antibacterial activity.

### 2.4. Determination of Antimicrobial Activity

Antimicrobial susceptibility test of the selected pathogens was done by Disc diffusion method using Kirby-Baeur technique [17] and as per recommendation of NCCLS [18]. All the tests were performed on Mueller Hinton agar plates. Suspension of microbial cultures (0.5 McFarlands) was inoculated on the entire surface of the Mueller Hinton agar media in a Petri plate using sterile swab sticks. The sterile discs of diameter 6 mm were impregnated with lichen extract solutions (0.1 mg/mL and 0.2 mg/mL) and placed onto the cultured Mueller Hinton agar plates. Inoculated plates were incubated at 37°C for 24 hrs.

On the second day, plates were read by taking measurement of zone of inhibition around each disc. The diameter of zone of inhibition of bacteria was recorded in millimeters. Pure acetone, methanol, and ethanol were taken as negative control as in accordance with Sati and Joshi, 2011 [19], whereas commercial Gentamicin and Ceftriaxone were used as positive control as in accordance with Owolabi et al., 2007 [20]. Gentamicin was taken as positive control for gram positive bacteria and Ceftriaxone was used for gram-negative bacteria. The assay was done in triplicates and checked with the control plate. To determine the affectivity of lichen crude extracts at different volumes, two different concentrations of lichen crude extracts were taken on each paper disc, on every Petri plate.

### 2.5. Minimum Inhibitory Concentration

The minimal inhibitory concentration (MIC) of the crude extract was determined by microdilution techniques in Mueller Hinton Broth (MHB), according to National Committee for Clinical Laboratory Standard, USA Guidelines [21]. A series of two fold dilutions with concentrations ranging from 100 *μ*g/mL to 0.195 *μ*g/mL for methanol extract was used in the experiment against *S. aureus*, *S. faecalis*, and *B. cereus*. For *P. aeruginosa*, no dilutions were done because no activity was recorded below 200 *μ*g/mL. Twofold dilutions of extracts and components were prepared in Mueller Hinton broth (MHB) for bacterial cultures. The inoculates were prepared in the same medium at a density adjusted to a 0.5 McFarland turbidity standard colony forming units, and diluted 1 : 10 for the broth microdilution procedure. Then, 100 *μ*L of diluted extracts and 100 *μ*L of bacterial suspensions were dispensed in 96 well sterile microtiter plate. The microtiter plates were incubated at 37°C and MIC was determined after 24 h of incubation. The MIC was determined by establishing visible growth of the microorganisms. The boundary dilution without any visible growth was defined as the MIC for the tested microorganism at the given concentration. Untreated bacteria were taken as positive control and MHB was taken as negative control. All experiments were performed in triplicate. 

### 2.6. Interpretation of Results

The results of disc diffusion assay are expressed as mean ± SD of three replicates in each test.

## 3. Results

### 3.1. Disc Diffusion Assays

After the treatment had been applied and the inoculated plates were allowed to grow for 24 hours, the acetone extract and ethanol extract of *U. ghattensis* were showing activity against *Staphylococcus aureus, Bacillus cereus*, and *Pseudomonas aeruginosa* while no activity was found against *Streptococcus faecalis, Escherichia coli*, and *Salmonella typhimurium*.

Both concentrations of methanol extract (0.1 mg/mL and 0.2 mg/mL) were showing activity against all the gram-positive bacteria and one gram-negative bacteria. No activity was recorded against *Escherichia coli*, *Salmonella typhimurium*. The acetone extract inhibited growth of *B. cereus* with a mean zone of 23.9 ± 1.1 mm (0.2 mg/mL conc.) while ethanol extract of the lichen had the greatest effect on plates inoculated with *Bacillus cereus* with a mean zone of inhibition of 29.8 ± 0.6 mm at 0.2 mg/mL concentration. The acetone and methanol extract were showing equal inhibitory effect on *S. aureus* with a mean zone of inhibition 24.6 ± 0.5 mm and 24.7 ± 0.4 mm at 0.2 mg/mL concentration, respectively.

The methanol extract showed poor activity against *S. faecalis* with a zone of inhibition 8.3 ± 0.5 mm at a concentration of 0.1 mg/mL while the concentration 0.2 mg/mL was showing a zone of inhibition of 13.5 ± 0.8 mm. Ethanol extract showed greater effect on *P. aeruginosa* with a zone of inhibition of 12.3 ± 0.5 mm at a concentration of 0.2 mg/mL in comparison to acetone (8.4 ± 0.6 mm dia. Zone) methanolic extract (8.7 ± 0.4 mm). Although the extracts were not as effective as the commercial antibiotics Gentamicin and Ceftriaxone, they have potent antibacterial activity (Table 1).

### 3.2. Minimum Inhibitory Concentration

The MIC values of the extract related to the tested bacterial strains varied between 25 and 3.125 *μ*g/mL in case of gram-positive bacteria. The measured MIC value for the extract against *Staphylococcus aureus* was 6.25 *μ*g/mL while the MIC value against *Bacillus cereus* was found to be 3.125 *μ*g/mL. *Streptococcus faecalis* was also showing 25 *μ*g/mL MIC value. Against *Pseudomonas aeruginosa*, the MIC value noted was 200 *μ*g/mL. Positive control was showing growth of bacteria and negative control was clear and not showing any growth of bacteria (Table 2).

## 4. Discussion

The intensity of the antimicrobial effect depended on the type of extract, its concentration, and the tested microorganisms. The tested concentrations of all the three extracts were showing activity against all bacteria except *S. faecalis*, for which only methanol extract was showing trace activity. Against *P. aeruginosa*, 0.2 mg/mL concentration was showing activity while the concentration 0.1 mg/mL was ineffective.

Acetone and methanol extract was showing almost equal activity against* S.aureus* whereas ethanol extract was found to be more effective against *B. cereus* and* P. aeruginosa*. The reason for different sensitivity of bacteria can be found in different transparency of the cell wall [22]. The cell wall of the gram-positive bacteria consists of peptidoglycan (mureins) and teichoic acids; the cell wall of the gram-negative cells consists of lipopolysaccharides and lipoproteins [23, 24]. Most of the Parmelloid lichens exhibit strong antimicrobial activity [25–27].

According to Burkholder et al. [28], Rowe et al. [29], and Silva et al. [30], the lichens inhibit mostly gram-positive bacteria, but it is of great interest to note that the extracts of *U. ghattensis* inhibited the growth of both gram-positive bacteria and one gram-negative bacteria in the present study.

*U. ghattensis* showed that MIC values were varying between 25 and 3.125 *μ*g/mL. Similar to other *Usnea* species, *U. ghattensis* also showed equal MIC values [31].

Lichens and their metabolites have manifold biological activity: antiviral, antibiotic, enzyme inhibitory, and allergenic. Behera et al. [32] reported that the acetone, methanol, and light petroleum extracts of lichen were effective against *Bacillus licheniformis*, *B. megaterium*, and *S. aureus*. Karagoz et al. [33] reported antibacterial activity of aqueous and ethanolic extracts lichens like *Lecanora muralis*, *Peltigera polydactyla*, *Ramalina farinacea*, and *Xanthoria elegans*.

## 5. Conclusion

The acetone, methanol, and ethanol extracts of *U. ghattensis* have a potential towards antibacterial activity. The obtained results showed that the tested lichen extracts showed a significant antimicrobial activity relative to the tested bacteria, which could be of significance in human therapy, animal, and plant diseases. Further investigations on the antibacterial activity as well as the economical and fast isolation of the metabolite from the lichen are needed. Consequently, the antibacterial effect of plants tested can be explained with new studies by using different solvents for extraction and other bacteria accurately. 

## Figures and Tables

**Table 1:** Zone of inhibition (mm) of extracts of *Usnea ghattensis *against tested microorganisms.

Serial number	Bacterial pathogen	Bacterial strain no.	Acetone extract		Methanol extract		Ethanol extract		Positive control	Negative control
			0.1 mg/mL	0.2 mg/mL	0.1 mg/mL	0.2 mg/mL	0.1 mg/mL	0.2 mg/mL	(Gentamicin and Ceftriaxone)	(pure solvent)
(1)	*Staphylococcus aureus *	ATCC 25923	13.8 ± 0.7	24.6 ± 0.5	15.2 ± 0.9	24.7 ± 0.4	8.8 ± 0.8	19.1 ± 0.8	25.6 ± 0.7	0.0 ± 0.0
(2)	*Streptococcus faecalis *	ATCC 33186	0.0 ± 0.0	0.0 ± 0.0	8.3 ± 0.5	13.5 ± 0.8	0.0 ± 0.0	0.0 ± 0.0	24.8 ± 0.6	0.0 ± 0.0
(3)	*Bacillus cereus *	ATCC 14579	20.6 ± 0.5	23.9 ± 1.1	19.1 ± 1.1	23.6 ± 0.5	20.4 ± 0.5	29.8 ± 0.6	29.1 ± 1.1	0.0 ± 0.0
(4)	*Escherichia coli *	ATCC 25922	0.0 ± 0.0	0.0 ± 0.0	0.0 ± 0.0	0.0 ± 0.0	0.0 ± 0.0	0.0 ± 0.0	25 ± 0.4	0.0 ± 0.0
(5)	*Pseudomonas aeruginosa *	ATCC 29853	0.0 ± 0.0	8.4 ± 0.6	0.0 ± 0.0	8.7 ± 0.4	0.0 ± 0.0	12.3 ± 0.5	26.6 ± 0.7	0.0 ± 0.0
(6)	*Salmonella typhimurium *	ATCC 13311	0.0 ± 0.0	0.0 ± 0.0	0.0 ± 0.0	0.0 ± 0.0	0.0 ± 0.0	0.0 ± 0.0	24 ± 0.6	0.0 ± 0.0

*Values are in mean ± standard deviation, *n* = 3.

**Table 2:** Results of Minimum Inhibitory Concentration (MIC) of extracts of *Usnea ghattensis* against tested microorganisms.

Serial number	Bacterial pathogen	Usnea ghattensis (MIC in *μ*g/mL)
(1)	*Staphylococcus aureus *	6.25
(2)	*Streptococcus faecalis *	25
(3)	*Bacillus cereus *	**3.125 **
(4)	*Escherichia coli *	NA
(5)	*Pseudomonas aeruginosa *	200
(6)	*Salmonella typhimurium *	NA

NA: no activity.
